# Cannabinoid Effect and Safety in Spasticity Following Stroke: A Double-Blind Randomized Placebo-Controlled Study

**DOI:** 10.3389/fneur.2022.892165

**Published:** 2022-06-23

**Authors:** Lucio Marinelli, Luca Puce, Laura Mori, Massimo Leandri, Gian Marco Rosa, Antonio Currà, Francesco Fattapposta, Carlo Trompetto

**Affiliations:** ^1^Department of Neuroscience, Rehabilitation, Ophthalmology, Genetics, Maternal and Child Health, University of Genoa, Genoa, Italy; ^2^Division of Clinical Neurophysiology, Department of Neuroscience, IRCCS Ospedale Policlinico San Martino, Genoa, Italy; ^3^Division of Neurorehabilitation, Department of Neuroscience, IRCCS Ospedale Policlinico San Martino, Genoa, Italy; ^4^Department of Internal Medicine, University of Genoa, Genoa, Italy; ^5^Cardiovascular Disease Unit, IRCCS Ospedale Policlinico San Martino, Genoa, Italy; ^6^Department of Medical-Surgical Sciences and Biotechnologies, A. Fiorini Hospital, Terracina, Sapienza University of Rome, Rome, Italy; ^7^Department of Human Neurosciences, Sapienza University of Rome, Roma, Italy

**Keywords:** Sativex, nabiximols, cannabinoid, pain, cerebrovascular disorders, blood pressure, THC, CBD

## Abstract

**Background:**

Nabiximols is a cannabis-based drug to treat spasticity-associated symptoms currently approved for patients with multiple sclerosis only. Cannabinoids are useful in an increasing number of medical conditions but may bear an increased risk for cardiovascular events. SativexStroke is a double-blind randomized placebo-controlled crossover monocentric clinical trial investigating the efficacy and safety of nabiximols in patients with spasticity following stroke.

**Methods:**

Patients were treated with nabiximols oromucosal spray or placebo and assessed before and after two phases of 1-month duration each. Cardiovascular safety was assessed before and during the trial. Primary endpoints were changes in spasticity numeric rating scale scores and electromyographic recording of the stretch reflex in affected wrist flexors. Secondary outcome measures were numeric rating scale scores for pain, sleep and bladder function, the number of daily spasms and clinical assessment of spasticity with the modified Ashworth scale. The study was registered with the EudraCT number 2016-001034-10.

**Results:**

Between May 2, 2018, and February 20, 2020, 41 patients entered the study. Seven patients did not complete the study, so 34 were included in the analysis. Two serious adverse events occurred, but none related to cardiovascular function. Primary and secondary efficacy outcome measures did not change from baseline during nabiximols treatment relative to placebo.

**Conclusion:**

This study suggests that nabiximols use is probably safe in stroke patients, therefore cannabinoid usefulness may be further investigated. The lack of nabiximols effect could be related to low pain levels in recruited patients or different spasticity mechanisms between post-stroke and multiple sclerosis patients. Similarly, a beneficial effect of nabiximols could have emerged if more patients with a higher level of spasticity at baseline were recruited.

**Clinical Trial Registration:**

https://www.clinicaltrialsregister.eu/ctr-search/trial/2016-001034-10/IT.

## Introduction

Stroke determines chronic consequences in about two-thirds of patients, including motor disability, speech impairment and limitations in basic tasks such as eating and self-care (1). Spasticity is a complication occurring in about 38% of stroke survivors within 12 months (2). The time interval between stroke and spasticity first appearance is variable, with about 25% of patients developing spasticity 2 weeks after stroke (1). In the first 3 months after stroke, the association between muscle hypertonia and severe motor impairment is an important predictor of the development of severe hypertonia 1 year after stroke (3). Spasticity refers to a pathological increase in muscle tone during stretching, that further impairs motor function and leads to permanent painful muscle contractures when untreated (4). Spasticity can produce pain and have a detrimental effect on daily functions, such as feeding, dressing, hygiene, and mobility (5). The presence of spasticity can have an emotional impact on mood, self-image and motivation. Spasticity increases care needs and utilization of health care and may extend further to affect work and productivity not only for patients but also for caregivers, resulting in a relevant cost for society (6). Botulinum toxin injection is considered the gold standard for the treatment of focal spasticity, however, it may be insufficient for those patients affected by spasticity affecting many muscles. Moreover, the effect lasts 3–4 months and therefore injections must be periodically repeated. When spasticity is severe and involves many muscles, drugs such as tizanidine, baclofen, thiocolchicoside, dantrolene and benzodiazepines may be prescribed, however, these drugs are little effective and hampered by many side effects and pharmacological interactions (7). Therefore, other systemic anti-spastic drugs with a different mechanism of action may be useful, either as an alternative or add-on to currently approved treatments. Importantly, the effect of anti-spastic pharmacological treatments depend on how much muscle hypertonia is due to reflex hypertonia (such as spasticity and spastic dystonia) rather than contracture. The latter is progressively more responsible for muscle hypertonia the more time passes following stroke, therefore reducing the effect of anti-spastic drugs.

Spasticity is due to increased excitability of stretch reflex spinal circuitry due to an imbalance between excitatory and inhibitory supraspinal drive. Anti-spastic effect of systemic drugs mainly occurs by increasing inhibitory or reducing excitatory neurotransmission (4). Although the mechanism of action of cannabinoids is still poorly understood, inhibition of presynaptic excitatory neurotransmitters release appears to be the main determinant (8).

Cannabinoids are attracting increasing interest as they are effective in treating pain, chemotherapy-induced nausea, appetite stimulation in wasting syndrome, epilepsy and spasticity (9, 10). Most of the studies of cannabinoid products in the treatment of spasticity-related symptoms were conducted in patients with multiple sclerosis, who are usually younger and with lower cardiovascular risk profile compared to stroke survivors (11–14). Phytocannabinoids consist of more than 60 different molecules coming from the Cannabis Sativa plant. There are also synthetic cannabinoids. They mainly bind to CB_1_ and CB_2_ endocannabinoid receptors, mimicking the endogenous human cannabinoids. The effects include inhibition of neurotransmitters release, synaptic plasticity and reward system modulation (15). CB_1_ receptors are primarily found in the central nervous system, but also in other organs and tissues, including endothelium. CB_2_ receptors are primarily expressed in peripheral tissues of the immune system. Concerning the central nervous system, CB_1_ and CB_2_ receptors show a higher expression in basal ganglia and cerebellum (16).

Several reports indicate an increased ischaemic stroke risk related to the abuse of smoked cannabis (marijuana) as well as synthetic cannabinoids (17). The “French Association of the Regional Abuse and Dependence Monitoring Centers Working Group on Cannabis Complications” warns about the increased cardiovascular risk related to the use of cannabis, mostly consisting of acute coronary syndromes and peripheral arteriopathies, potentially leading to life-threatening conditions (18). The detrimental consequences of high cannabinoid levels could be attributed to the increase in heart rate (19) as well as arterial spasm also in the context of a reversible cerebral vasoconstriction syndrome (20), but also vasculitis and cardioembolism (21).

On the other hand, studies in animal models support the beneficial effect of cannabinoid receptors stimulation. In fact, cannabinoid mediated activation of CB_1_ and CB_2_ receptors reduces inflammation and neuronal injury in acute ischaemic stroke models (22). Activation of CB_2_ receptors shows protective effects after ischaemic injury (23) and inhibits atherosclerotic plaque progression (24).

Nabiximols (Sativex®) is basically a combination of the two main cannabinoids delta-9-tetrahydrocannabinol (THC) and cannabidiol (CBD) in 1:1 ratio administered through the oromucosal route. This drug underwent an extensive clinical trial program (11–14) in adult patients with multiple sclerosis, supporting effectiveness on spasticity-associated symptoms and an acceptable safety profile, including cardiovascular safety parameters.

Our hypothesis is that, under controlled medical conditions, nabiximols could be useful also in stroke survivors without inducing relevant cardiovascular side effects. The aim of this double-blind randomized placebo-controlled crossover trial is to assess nabiximols safety and efficacy on spasticity in post-stroke patients.

## Methods

### Study Design

SativexStroke was a randomized double-blind, placebo controlled, crossover design of 10 weeks duration: phase one (4 weeks, from T0 to T1), washout (2 weeks, from T1 to T2) and phase two (4 weeks, from T2 to T3). The aim is to assess the effect of nabiximols on post-stroke spasticity. Allocation ratio was 1:1. The study was performed at the outpatient service for the treatment of spasticity of the Neurorehabilitation Unit, IRCCS Ospedale Policlinico San Martino (Genova, Italy) in accordance with the Declaration of Helsinki and Good Clinical Practice guidelines. The study protocol was approved by the local ethics committee and published in an international peer-reviewed journal (25). The full trial protocol is available from the corresponding author upon request. The trial was registered on the EudraCT platform with number 2016-001034-10.

Right before first patient entered the study, the original protocol underwent a major amendment. While originally the outcome measures at the end of phase 1 (T1) and phase 2 (T2, now T3) had to be compared to baseline condition (T0), in order to compensate for possible variability of the outcomes measured throughout the study, an additional visit was performed before phase 2 (T2), to obtain a new baseline condition to compare with the last visit (T3). No other substantial variation from the original protocol occurred during the study.

### Participants

Adult stroke survivors were recruited according to the following inclusion criteria: (1) male or female patients of at least 18 years of age; (2) spasticity secondary to stroke that occurred at least 3 months earlier; (3) CHA_2_DS_2_VASc score <7 assessed by the cardiologist and reflecting acceptable cardiovascular risk; (4) spasticity rated between 1 and 3 on the Modified Ashworth Scale at the level of at least one of the following muscle groups: wrist flexors, elbow flexors, knee extensors, foot plantar flexors; (5) able (physical ability or supportive person) to comply with the study requirements correctly and to follow the study procedure and restrictions.

Exclusion criteria were: (1) presence of concomitant parkinsonism; (2) significant peripheral nervous system pathology detectable on clinical basis; (3) current smokers; (4) contraindication to treatment with nabiximols; (5) alcohol or drug abuse, including current consumption of cannabis herb or other cannabinoid-based drugs within 30 days prior to study entry; (6) treatment with botulinum toxin injection in the last 4 months; (7) clinically significant impaired renal function or impaired hepatic function at baseline, (8) females of child bearing potential, pregnant or lactating and male patients whose partner is of child bearing potential who are not willing to use effective contraception; (9) absence of significant cognitive impairment hampering patients' capability of understanding the study protocol and signing the consent form.

### Patient and Public Involvement

Patients, families and the public were not directly involved in the study planning and assessment of the consequent burden of the intervention and time required to participate in the research.

Most of the patients who participated in the study were assisted by the recruiting neurorehabilitative outpatient service, indirectly providing useful information about their therapeutic unmet needs and logistic issues that strongly influenced the planning of the study. Furthermore, during the recruitment phase, two articles explaining the aim of the study have been published in online newsletters accessible to both healthcare professionals and the general public.

### Randomization

Eligible subjects were randomized in a 1:1 allocation to one of two treatment sequences: nabiximols-placebo or placebo-nabiximols by means of a randomization list generated through a validated SAS® program. Block randomization with 4 patients per block (2 nabiximols and 2 placebo) was used to reduce bias and to achieve balance in the allocation of participants to treatment arms in the study. All individuals involved in the study conduct, including patients, investigator staff, persons performing the assessments and data analysts remained blinded to the identity of the treatment from the time of randomization until database lock. Being a pilot study, no a priori sample size calculation was done.

### Procedures

**Figure 1:**
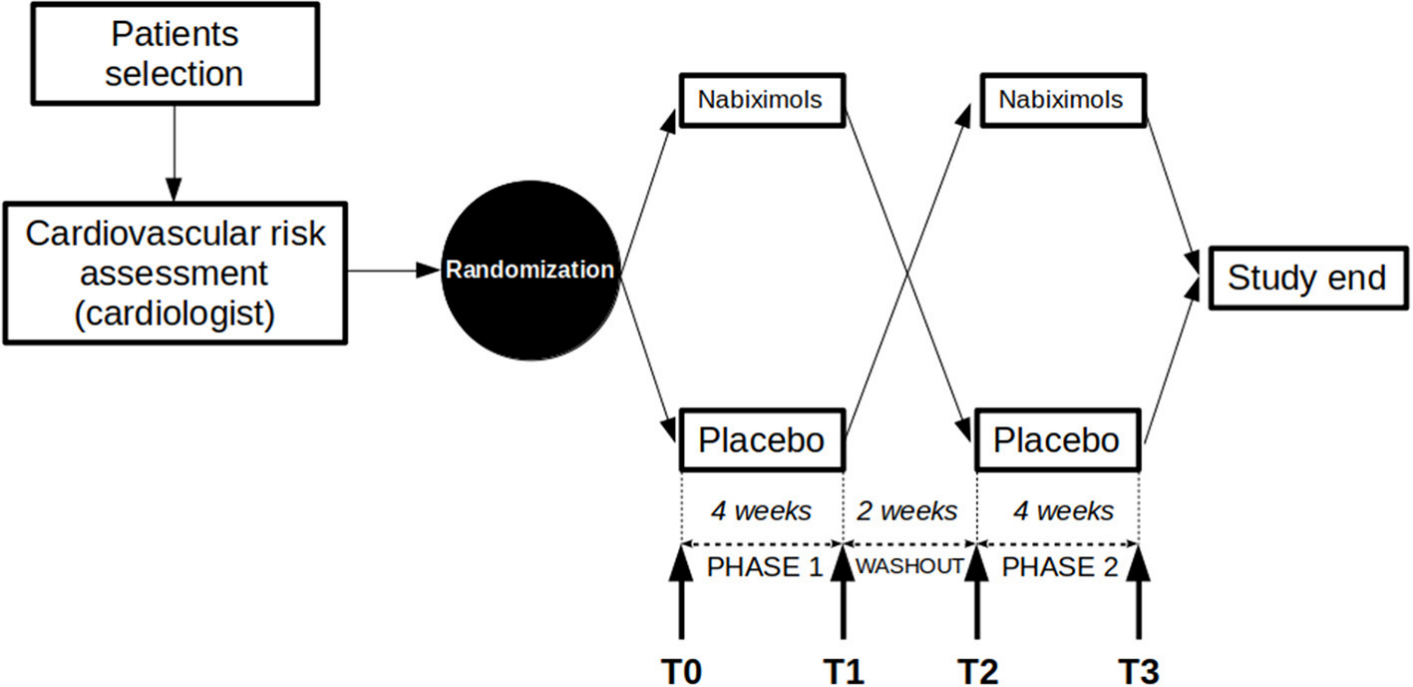
Study protocol design. Study crossover design is represented. After checking for inclusion/exclusion criteria and passing the cardiological evaluation, patients were randomized to placebo/nabiximols or nabiximols/placebo arms. The two phases of the study were separated by a washout period, started with visits T0/T2 and ended with visits T1/T3.

Patients who gave informed consent to participate underwent a preliminary screening visit to ensure that they fulfilled the study selection criteria, followed by a cardiological evaluation (including ECG and echocardiogram) to assess cardiovascular risk. Once deemed eligible, patients were randomized and entered the study, consisting of two phases separated by a wash-out period (2 weeks), following a cross-over design. Each phase lasted 4 weeks and was preceded and followed by a visit (4 study visits in total: T0 and T1 for phase 1, T2 and T3 for phase 2), so for each patient the study lasted 10 weeks (Figure 1). All participants were informed about the most frequently expected adverse events (dizziness, tiredness, feeling drunk, instability, etc.) and provided with the mobile phone number of the principal investigator to promptly report any unexpected major adverse event and allow consultation at any time during the study.

At baseline evaluation (T0), patients were provided with a form to daily record heart rate, blood pressure and adverse events. Outcome measures were collected and instructions on how to take the oromucosal spray were provided, following the instructions reported into the official Sativex patient leaflet. Specifically, patients were instructed to gradual increase daily sprays following a printed schedule (one spray every 2 days for the first 4 days, than increase one spray per day) to reach the highest tolerable dose up to a maximum of 12 sprays/day (divided into two administrations: morning and evening) in a 14-days period and then maintain such daily dose until the end of the first phase (T1). Patients were required to continue their concomitant anti-spastic medications (baclofen, tizanidine, benzodiazepines, etc.) or other drugs with potential effect on spasticity (gabapentin, tricyclic antidepressants, painkillers, etc.) throughout the study. Similarly, patients were required to keep unchanged their current rehabilitation treatment and level of physical activity.

At T1, patients underwent scheduled assessment including recording of adverse events, daily blood pressure and heart rate. Study drug vials were collected in order to start the wash-out phase and return to baseline conditions before starting the second phase of the study.

T2 and T3 evaluations, respectively, opened and closed the second phase of the study, with patients taking the other assigned study drug following the same up-titration protocol.

### Outcomes

Outcome measures were collected during all 4 study visits. Stretch reflex was assessed on a spastic muscle segments preferring in order: wrist flexors, elbow flexors, knee extensors, foot plantar flexors. The modified Ashworth scale score (MAS, 0–4 clinical rating scale) in the same selected muscle group was also separately considered for analysis (selectMAS). Dual primary outcome measures were: (1) stretch reflex mean amplitude in the selected muscle group (meanEMG) and (2) numeric rating scale for spasticity (0–10) (spasNRS). Patients were asked to report the effect on other aspects of upper motor neuron syndrome, such as pain, spasms, bladder function and their impact on sleep quality. Consequently, we obtained a subjective rating of these symptoms as secondary outcome measures: (1) subjective amount of pain (0–10 numeric rating scale) (painNRS), (2) daily spasms count (spasmN), (3) sleep quality (0–10 numeric rating scale) (sleepNRS), (4) bladder dysfunction (0–10 numeric rating scale) (bladNRS), (5) selectMAS, (6) sum of MAS scores in all evaluated segments (0–44 clinical rating scale) of the affected side (totalMAS). Higher values in NRS always indicated more severe symptoms; daily spasm count indicates the number of spasms reported by the patients during the previous 24 h. Patients were instructed to recognize spasms as involuntary transient muscle contractions that cause one or more limbs to flex or extend. Similarly, all subjective scores (spasNRS, painNRS, sleepNRS and bladNRS) reflected estimated average level of symptoms during the 24 h preceding each scheduled visit. Higher MAS scores indicate greater resistance to passive stretch assessed by the clinician through joint mobilization.

Safety and tolerability outcomes included the number of patients with adverse events and these with serious adverse events, the number of treatment interruptions related to adverse events including significant alterations of both systolic/diastolic blood pressure and heart rate. Cardiological evaluation and ECG were not routinely repeated during the study.

The totalMAS score was computed in addition to the secondary outcomes established in the original protocol, in order to evaluate the effect on spasticity not limited to a single muscle but on multiple limb segments. The totalMAS score was therefore calculated as the sum of MAS scores of 11 muscle groups on the affected body side: shoulder adductors, elbow flexors, elbow extensors, forearm pronators, wrist flexors, fingers flexors, thigh adductors, knee flexors, knee extensors, ankle plantar flexors, foot supinators.

### Stretch Reflex Recording

The stretch reflex corresponds to the electromyographic activity generated by a muscle while passively stretched. Lesions affecting the central motor pathways, as in stroke, often decrease the inhibitory drive on spinal circuitry responsible for myotatic reflexes, so that the excitability of the stretch reflex increases. Passive elongation of the affected muscles therefore induces a reflex activation at lower velocities and of higher amplitude compared to normal subjects. The hyperexcitability of the stretch reflex represents the mechanism underlying spasticity (4, 26); therefore, electromyographic recording of the stretch reflex is an important measure of spasticity.

Subjects were evaluated in supine position in a quiet and warm room. Electromyographic activity of the selected muscle group (flexor carpi radialis, since all patients had a selectMAS score of at least 1 on wrist flexors, see Results) on the affected side during stretches was recorded by adhesive surface electrodes (Neuroline 700, Ambu A/S, Ballerup, Denmark) placed over the muscle belly with 2 cm inter-electrode distance. In order to minimize variability related to different electrode positioning on the muscle, a picture was taken at T0 and during consequent visits the electrodes were repositioned according to the picture. Wrist joint angle during passive stretches was also acquired by means of an electronic goniometer (TSD130B, Biopac Systems Inc, USA). A low-noise amplifier (BIOAMP LT, Vertigo, Genova, Italy) acquired both signals, that underwent analog to digital conversion and storage for offline analysis.

Since spasticity is a velocity-dependent phenomenon, the amplitude of the stretch reflex depends on passive movement velocity. Reproducible wrist movement velocities are therefore crucial in order to compare the effect of treatments capable of reducing stretch reflex amplitude. In order to easily obtain reproducible passive wrist movements, we used a method validated in previous studies (27). The method consists of moving the patient's wrist throughout the full range of motion reaching maximal flexed and extended positions in synchrony with consecutive metronome tones. Setting a higher or lower tone frequency (beats per minute) determines faster or slower movements, respectively. Waiting a few (1–4) tones before performing the subsequent flexion or extension movement renders the movements discontinuous, allowing a more precise separation of the electromyographic activity during consecutive movements.

At T0, for each patient the optimal passive movement timing was decided based on the amount of baseline spasticity: in patients with less spasticity, higher velocities were needed to record stretch reflexes, while in patients with higher spasticity, stretch reflexes were clearly recordable at lower velocities. Consequently, for each patient the appropriate velocity was selected al T0 and used for all subsequent evaluations. In each visit, 20 consecutive stretch reflexes were recorded from the affected flexor carpi radialis muscle. High-pass filter at 50 Hz was applied before measuring the rectified average amplitude of the electromyographic signal during wrist extension passive movements. The mean value of the first 10 stretches was considered as the stretch reflex amplitude (meanEMG) for analysis purpose.

### Statistical Analysis

Clinical data were extracted from the case report forms and stretch reflex amplitude from the electromyographic recordings. The resulting database underwent a preliminary check and clean-up by OPIS S.r.l. (Desio, Italy). They provided concomitant medication coding at the beginning of the study and conducted database lock and unblinding at the end of the study.

Sequence and period effects on outcomes were analyzed to investigate any carry-over effect.

**Figure 2:**
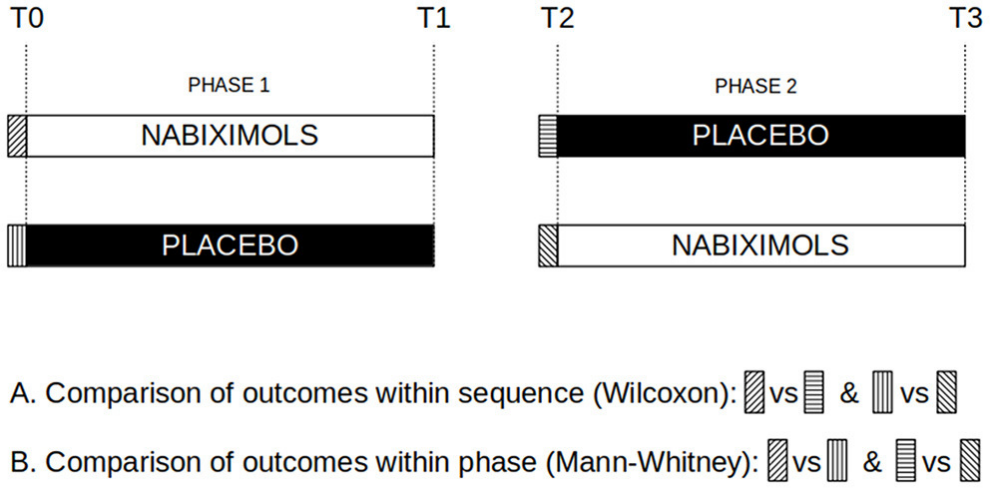
Risk of bias assessment. Graphical representation of assessments to compares outcomes within sequence (T0 vs. T2) and within phase (nabiximols vs. placebo at T0 and T2).

Firstly, a Wilcoxon signed rank test was applied to compare outcome between treatments within sequence. Secondly, a Mann-Whitney test was applied to compare outcome within phase (Figure 2).

The following variables underwent statistical analysis: spasNRS, meanEMG (dual primary outcomes), painNRS, spasmN, sleepNRS, bladNRS, selectMAS, totalMAS (secondary outcomes). All variables were compared before (T0/T2) and after (T1/T3) active and placebo treatments in the 34 patients that completed the study using Wilcoxon signed rank test. Furthermore, variables across matched time points (T1-T0 and T3-T2 differences) were compared between active and placebo treatments using Wilcoxon signed rank test, in order to tell apart the effect of nabiximols from that of placebo.

Mann-Whitney test was used for baseline comparison of age and time after stroke between treatment sequence groups starting with nabiximols and placebo.

Based on published literature on nabiximols effect on spasticity, responders were defined as those patients showing a 20 or 30% spasNRS improvement from baseline. McNemar's test was used to compare the responders rate between active and placebo treatments.

In order to disclose a possible effect of nuisance variables (age, time passed since stroke, time since last botulinum toxin injection and number of sprays/day) on primary outcomes, spasNRS and meanEMG variation during active treatment was correlated with these nuisance variables using Spearman rank correlation test.

Differences were considered significant when *p* < 0.01. Measures are reported as median and interquartile range (Q1–Q3) or mean ± standard deviation. All analyses were performed using StatView version 5.0 (SAS Institute).

While statistical analyses were performed only on the 34 patients who completed the study, safety data related to adverse events and concomitant medications included all 41 patients who entered the study.

## Results

### Recruitment and Participant Flow

**Figure 3:**
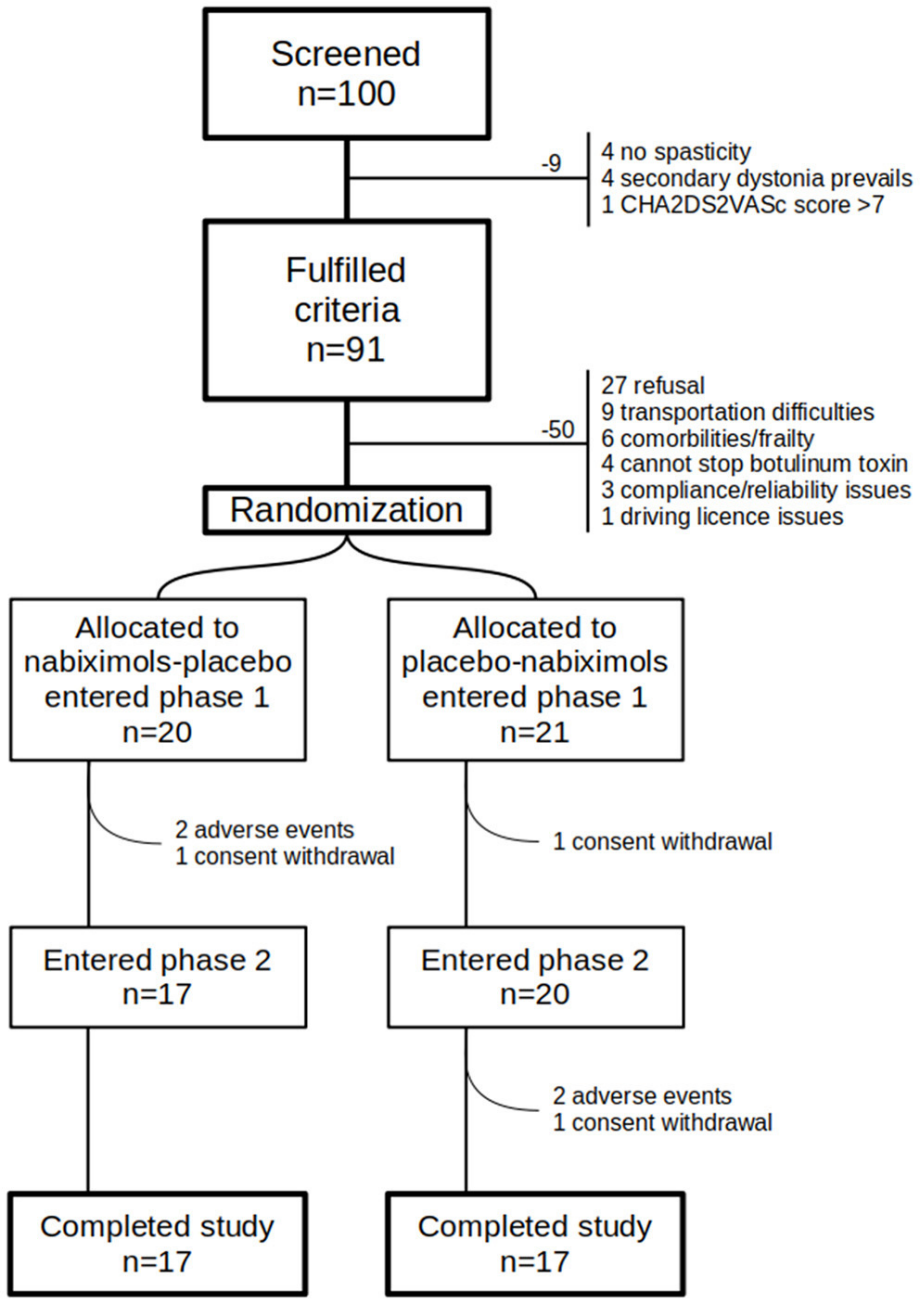
Participants flow diagram. Graphical representation of patients flow. Many patients fulfilled inclusion/exclusion criteria but had to be excluded because of many reasons, mainly refusal.

Among 100 pre-screened patients, 91 met inclusion criteria and were considered for study participation, however 50 patients did not enter the study for the following reasons: refusal to participate (27), transportation difficulties (9), medical decision due to relevant comorbilities or frailty (6), medical decision due to excessive spasticity and need to continue botulinum toxin (4), compliance/reliability issues (3), fear for driving license suspension (1) (Figure 3). Finally, 41 patients signed the consent form and started the study. Since 7 patients exited the study (see below), 34 patients completed all assessments and were included in the primary analysis set (Table 1). Recruitment started on May 2nd, 2018 (first patients in) and follow-up ended on February 20th, 2020 (last patient out).

**Table 1:** Baseline patient characteristics.

	**Nabiximols-placebo**	**Placebo-nabiximols**	Total^*^
	***N* = 17**	***N* = 17**	***N* = 34**
**Gender**			
Male	10 (59%)	14 (82%)	24 (71%)
Female	7 (41%)	3 (18%)	10 (29%)
Age (y)	65 (58–73)	69 (61–71)	68 (59–72)
**Stroke type**			
Hemorrhagic	7 (41%)	6 (35%)	13 (38%)
Ischemic	10 (59%)	11 (65%)	21 (62%)
**Affected side**			
Right	6 (35%)	7 (41%)	13 (38%)
Left	11 (65%)	10 (59%)	21 (62%)
Time after stroke (y)	2.6 (1.3–4.9)	4.9 (2.7–10.1)	4.2 (1.7–5.6)

* *Patients who completed the study (both phase 1 and phase 2). Values are reported as number (%) or median (Q1–Q3)*.

### Adverse Events and Patients Withdrawals

There were 7 withdrawals: 4 during the first phase and 3 during the second phase of the study. Four patients withdrew because of side effects (2 during the first phase, 2 during the second phase; all 4 while taking nabiximols) and 3 because of study consent withdrawal (2 during the first phase, 1 during the second phase: 1 was taking placebo, 2 were taking nabiximols).

No significant blood pressure and heart rate variation occurred in any patient. No antihypertensive drug dosage adjustment or additional cardiological consultation was required.

All non-serious adverse events can be considered mild, expected and mostly related to the study drug. Those causing patients withdrawal were: subjective motor worsening, tachycardia, sleepiness, confusion, fatigue and nausea (Table 2).

**Table 2:** Complete list of adverse events.

**N**	**Gender**	**Age**	**Status**	**Drop reason**	**SAE**		**AE-1 phase**		**AE-2 phase**
1	M	71	Completed		No	N	None	P	Confusion, diarrhea
2	M	72	drop1	AE	No	N	Subjective motor worsening	P	–
3	M	61	Completed		No	P	Seborrhoeic dermatitis worsening	N	Seborrhoeic dermatitis worsening
4	F	55	Completed		No	P	None	N	Weakness, dizziness above 2 puff/day
5	M	69	Completed		No	P	Thirst	N	Thirst, weakness, fatigue, pain, fear of falling
6	F	74	Completed		No	N	None	P	None
7	M	60	Completed		No	N	Nausea, pharyngodynia, malaise, sweating	P	Morning sleepiness, nausea, dizziness
8	M	70	drop2	compliance	No	P	None	N	None
9	M	71	Completed		No	P	None	N	Dizziness, tiredness, feeling drunk, night urinary incontinence, slowness
10	F	76	Completed		No	N	Dizziness, tiredness	P	Headache
11	M	68	Completed		No	N	None	P	None
12	M	61	drop1	compliance	No	P	None	N	–
13	M	44	drop2	AE	No	P	Constipation, tachycardia	N	Tachycardia
14	M	74	Completed		No	N	None	P	None
15	M	56	Completed		No	P	None	N	None
16	M	58	Completed		No	N	Feeling drunk, instability, tinnitus, dizziness	P	None
17	M	51	drop1	AE	No	N	Sleepiness, confusion, fatigue	P	–
18	M	81	Completed		No	P	None	N	Sleepiness
19	M	65	Completed		No	P	None	N	Dizziness, nausea, feeling drunk
20	F	59	Completed		No	N	Dizziness, feeling drunk	P	None
21	M	62	Completed		No	P	None	N	Dizziness, feeling drunk
22	M	64	Completed		No	N	Dizziness, confusion, malaise	P	Mild esophageal reflux
23	M	70	Completed		No	P	None	N	None
24	F	68	Completed		No	N	Dizziness, instability while walking	P	Instability while walking, weakness, depression
25	F	72	Completed		No	P	Dizziness	N	Dizziness, feeling drunk, lower limb weakness
26	M	56	Completed		No	P	None	N	Sleepiness, confusion
27	F	65	Completed		No	N	None	P	Confusion, incontinence
28	M	45	Completed		No	N	Confusion, incontinence	P	None
29	M	70	Completed		No	P	None	N	Dizziness, visual allucinations
30	F	49	Completed		No	N	Sleepiness	P	None
31	F	82	Completed		No	N	None	P	None
32	M	72	Completed		No	P	Visual allucinations	N	Sleepiness, instability while walking, dizziness, headache, seborrheic dermatitis worsening
33	M	54	drop2	AE	Yes	P	None	N	Nausea
34	M	58	Completed		No	N	Anxiety	P	Visual fogging, forearms erythema
35	M	68	Completed		No	P	Sleepiness	N	Sleepiness, weakness, dizziness
36	M	73	Completed		No	N	Instability while walking, weakness, difficulty concentrating	P	None
37	M	55	Completed		No	N	Sleepiness, dizziness, nausea	P	None
38	M	76	Completed		No	P	Sleepiness, cramps, increased limb stiffness	N	Tiredness, weakness, difficulty walking
39	M	58	drop1	compliance	No	N	None	P	–
40	F	61	Completed		Yes	P	Nausea, dizziness	N	Epileptic seizure, dizziness, nausea
41	M	70	Completed		No	P	Weakness, fatigue, shortness of breath	N	Shortness of breath

*AE, adverse events; CW, consent withdrawal; SAE, serious adverse events; P, placebo, N, nabiximols*.

Only two patients reported serious adverse events while taking nabiximols. Patient number 33 presented with severe nausea and went to the emergency room without contacting the principal investigator. Nausea revolved completely following study drug suspension with no consequences. Even if nausea is an expected adverse event, overnight hospitalization renders this drug-related adverse event as serious, even if no complications occurred and the patient recovered promptly. The second serious adverse event occurred in patient number 40. She presented a first generalized epileptic seizure and was admitted to the emergency room, where levetiracetam 1 g/day was started. Following additional investigations, the seizure was considered related to the existing brain lesion following the stroke and unrelated to the study drug. The patient continues the study, which she completed without reporting other adverse events.

### Concomitant Medications

Patients were required to continue taking concomitant medications at the same dosage throughout the study. Among the 41 included patients, 17 (41%) were taking medications with anti-spastic effect: baclofen in 11 patients (27%, mean dosage 36.8 mg/day), pregabalin in 2 (5%, mean dosage 137.5 mg), zolpidem in 2 (5%, 10 mg in both), triazolam in 2 (5%, 0.25 mg in both), lorazepam in 2 (5%, mean dosage 1.9 mg), gabapentin in 2 (5%, mean dosage 750 mg), alprazolam in 1 (0.125 mg), oxazepam in 1 (30 mg), diazepam in 1 (3 mg) and clonazepam in 1 (0.2 mg).

### Effect on Outcome Measures

The median number of sprays taken was 12 (11–12) in patients on placebo treatment at the end of the first phase (T1) and 12 (10–12) in patients on placebo treatment at the end of the second phase (T3). The median number of sprays was 10 (7–12) in patients on nabiximols at the end of the first phase (T1) and 8 (3–12) at the end of the second phase (T3). Taken together, the 34 patients were taking on average 12 (10–12) sprays of placebo and 9 (4–12) sprays of nabiximols.

Numeric rating scale for spasticity (spasNRS) did not change from baseline either after nabiximols (*p* = 0.017) or placebo (*p* = 0.028); the two treatments did not differ at matching time points (*p* = 0.7) (Table 3).

**Table 3:** Primary and secondary outcomes.

**Primary outcomes**	**T0/T2**	**T1/T3**	**p**
Spasticity NRS			**0.7**
Nabiximols	5.8 ± 2.2	5.1 ± 2.0	0.02
Placebo	5.7 ± 2.1	5.2 ± 2.2	0.03
meanEMG			**0.2**
Nabiximols	19.8 ± 12.8	21.3 ± 14.2	0.3
Placebo	21.1 ± 13.7	20.1 ± 11.8	0.1
**Secondary outcomes**	**T0/T2**	**T1/T3**	**p**
Pain NRS			**0.2**
Nabiximols	2.4 ± 2.8	2.1 ± 2.6	0.3
Placebo	2.5 ± 2.6	1.9 ± 3.2	0.005
Spasm number			**0.4**
Nabiximols	1.1 ± 2.2	1.3 ± 2.6	0.9
Placebo	1.2 ± 2.3	0.9 ± 2.1	0.04
Sleep NRS			**0.05**
Nabiximols	3.3 ± 2.9	2.7 ± 2.9	0.04
Placebo	2.7 ± 3.3	2.8 ± 3.2	0.2
Bladder NRS			**0.7**
Nabiximols	3.1 ± 3.0	3.0 ± 3.0	0.5
Placebo	2.8 ± 3.0	2.8 ± 3.0	0.5
Wrist flexors MAS			**0.05**
Nabiximols	2.1 ± 0.7	2.0 ± 0.8	0.1
Placebo	2.1 ± 0.7	2.1 ± 0.9	0.8
Total MAS			**0.9**
Nabiximols	15.3 ± 6.0	13.8 ± 5.5	0.01
Placebo	15.7 ± 5.4	14.4 ± 6.2	0.03

*All measures are reported as mean ± standard deviation. p-values represent difference between treatments (bold) and the effect of each treatment compared to baseline (T1/T3 vs. T0/T2). NRS, Numeric Rating Scale; MAS, Modified Ashworth Scale: lower scores indicate less impairment*.

Published literature defines responders to the treatment with nabiximols as assessed with spasNRS as those patients who show a 20 or 30% improvement of spasNRS compared to baseline values. In our study, the number of patients with at least 20% spasNRS improvement was 7 (41.2%) for placebo/phase 1, 3 (17.6%) for placebo/phase 2, 6 (35.3%) for nabiximols/phase 1 and 5 (29.4%) for nabiximols/phase 2. Overall, 20% responders were 10/34 for placebo (29.4%) and 11/34 for nabiximols (32.4%). The number of patients with at least 30% spasNRS improvement were 2 (11.8%) for placebo/phase 1, 1 (5.9%) for placebo/phase 2, 3 (17.6%) for nabiximols/phase 1 and 1 (5.9%) for nabiximols/phase 2. Overall 30% responders were 3/34 for placebo (8.8%) and 4/34 for nabiximols (11.8%). McNemar's test did not disclose a significant difference in the number of responders between nabiximols and placebo considering either 20 and 30% cutoffs.

Since spasticity was detected on wrist flexor muscles in all the patients, this muscle group was always selected to obtain meanEMG and selectMAS values. A stretch reflex in flexor carpi radialis could be elicited in all subjects during all 4 assessments. MeanEMG did not change from baseline either after nabiximols (*p* = 0.3) and placebo (*p* = 0.1) treatments; no significant difference also emerged between treatments across matching time points (*p* = 0.2) (Table 3).

During nabiximols treatment, no significant correlation emerged between changes in primary outcomes (spasNRS and meanEMG) and possible nuisance variables such as age, number of sprays/day, time since last botulinum toxin injection and time since stroke date.

Considering secondary outcomes, painNRS significantly decreased from baseline after placebo (*p* = 0.005) but not after nabiximols (*p* = 0.3) and the two treatment did not differ at matching time points (*p* = 0.2). No significant changes from baseline following nabiximols or placebo treatment emerged for the other secondary outcomes (spasmNRS, sleepNRS, bladNRS, selectMAS, totalMAS) (Table 3).

### Risk of Bias Assessment

Age (*p* = 0.6) and time after stroke (*p* =0.09) did not differ between the two groups. Risk of bias was assessed by comparing primary outcomes across nabiximols-placebo and placebo-nabiximols sequences (Table 4, part A) as well as across phase 1 (T0–T1) and phase 2 (T2–T3) (Table 4, part B). No significant differences emerged for either comparisons.

**Table 4:** Risk of bias assessment.

**(A) Comparison of primary outcomes within sequence**						
	**Sequence Nabiximols-Placebo**			**Sequence Placebo-Nabiximols**		
	**Nabiximols**	**Placebo**	***p*** -value^*^	**Placebo**	**Nabiximols**	***p*** -value^*^
	**(Phase 1)**	**(Phase 2)**		**(Phase 1)**	**(Phase 2)**	
	**(*****N*** **= 17)**	**(*****N*** **= 17)**		**(*****N*** **= 17)**	**(N=17)**	
spasNRS	6.8 ± 1.7	6.3 ± 1.8	0.1	5.2 ± 2.3	4.8 ± 2.1	0.2
meanEMG	18.9 ± 11.7	20.9 ± 12.1	0.2	21.4 ± 15.5	20.7 ± 14.1	0.9
**(B) Comparison of primary outcomes within phase**						
	**Phase 1**			**Phase 2**		
	**Nabiximols**	**Placebo**	***p*** -value^**^	**Nabiximols**	**Placebo**	***p*** -value^**^
	**(*****N*** **= 17)**	**(*****N*** **= 17)**		**(*****N*** **= 17)**	**(*****N*** **= 17)**	
spasNRS	6.8 ± 1.7	5.2 ± 2.3	0.03	4.8 ± 2.1	6.3 ± 1.8	0.04
meanEMG	18.9 ± 11.7	21.4 ± 15.5	0.8	20.7 ± 14.1	20.9 ± 12.1	1.0

* *p-value for Wilcoxon signed rank test. All values are reported as mean ± standard deviation*.

** *p value for Mann-Whitney U-test. All values are reported as mean ± standard deviation*.

## Discussion

### Cannabinoid Safety in Post-stroke Patients

The present study is the first to ascertain the effect of a cannabinoid-based medication in patients with spasticity following stroke since previous studies have been performed only on animal models of stroke spasticity (28). Nabiximols is a combination of delta-9-tetrahydrocannabinol and cannabidiol in a balanced ratio as well as other cannabinoid and non-cannabinoid components, administered via oromucosal route to treat spasticity. It has been widely studied in patients with spasticity due to multiple sclerosis (11–14) and also in one randomized controlled trial in patients with amyotrophic lateral sclerosis (29), but not for the treatment of spasticity in stroke survivors, where there may be a substantial unmet need given the high stroke prevalence (2). This is probably due to the high-risk cardiovascular profile of patients who had a stroke, along with previous reports warning about higher stroke risk in subjects with cannabinoid abuse (17). To our knowledge, nabiximols-related stroke events have not been reported in the literature so far. Published case reports or case series of stroke were related to smoked natural or synthetic cannabinoid (17). A much higher proportion of THC compared to CBD along with the higher rate and extent of exposure to THC and its metabolites in plasma following administration of cannabis by inhalation (30) could possibly play a role in facilitating blood pressure fluctuations in patients with compromised cerebrovascular circulation and ensuing stroke (20). In our study, all patients underwent a preliminary cardiological evaluation (including ECG and echocardiogram) and daily monitored blood pressure and heart rate. Such parameters did not change significantly during the study and no patient presented cardiovascular complications. Only one patient (number 13) complained about tachycardia and decided to exit the study for this reason (during phase 2 under active treatment), even if tachycardia was already present during phase 1, while taking placebo. Six patients were not recruited because of medical comorbidities and frailty mainly consisting of chronic respiratory distress, impaired renal function or neoplasm, indeed none of these comorbidities was cardiovascular in nature.

Overall, nabiximols was well-tolerated, common adverse events were largely expected and mainly consisted in dizziness and confusion. Only two serious adverse events occurred, both during active treatment. The first resulted in overnight admission to the hospital because of a common side effect (nausea and vomiting) that was not reported to the principal investigator in the first place. The second resulted in hospitalization because of an epileptic seizure, which was considered unrelated to the drug. This patient decided to continue in the study, which was concluded without other problems. No patient presented a new stroke or other cardiovascular adverse events. Therefore, the present findings suggest that nabiximols is safe in stroke patients with spasticity. However, it must be acknowledged that the present study included only 41 subjects, dosing duration was limited to 4 weeks and cardiovascular monitoring during the study included only daily blood pressure and heart rate measurements.

### Cannabinoid Effect on Outcome Measures

The present study does not suggest an improvement in spasticity from the treatment of patients with post-stroke spasticity with nabiximols. No significant improvement of subjective (spasNRS), clinical (selectMAS, totalMAS) and neurophysiological (meanEMG) measures of spasticity emerged during active treatment compared to placebo. It is worth underlining that electromyographic recording of stretch reflex (meanEMG) to measure spasticity is particularly appropriate since stretch reflex increase represents the actual mechanism underlying spasticity (26). It can be quantitatively and objectively measured and the placebo effect is negligible. Using a similar methodological approach in an open-label, before-and-after study with a comparable number of patients affected by multiple sclerosis, we observed a significant reduction of spasNRS, MAS and meanEMG (31).

The apparent lack of nabiximols effect on spasticity observed in this limited sample of patients with post-stroke spasticity could be related to the partially different mechanisms underlying spasticity in stroke and multiple sclerosis (32). While in patients with multiple sclerosis spasticity is often generated by lesions located both in the brain and spinal cord, in all our post-stroke patients lesions were confined to the brain (33). Moreover, it is well known that spasticity can be enhanced by pain and vice-versa. Therefore, pain reduction may improve spasticity as a consequence (4). In our post-stroke patients, baseline (T0/T2) pain NRS level was low (2.4) and was not affected by the active treatment or placebo, while in our previous open-label study in patients with multiple sclerosis pain level was higher (3.9) and improved significantly after initiation of treatment with nabiximols (31). Nevertheless, the present findings, in stroke patients with low levels of pain showing a lack of effect of nabiximols on spasticity are important since they suggest that nabiximols does not directly modulate spinal circuitry responsible for spasticity. Accordingly, using another neurophysiological approach to assess the spinal mechanisms underlying spasticity (H reflex), nabiximols did not produce a significant effect in patients with multiple sclerosis (32, 34).

Patients with spasticity due to stroke or multiple sclerosis differ also in other features. Stroke survivors are usually older than those with multiple sclerosis and the degree of upper motor neuron impairment stabilizes during the weeks following the acute lesion. Conversely, in multiple sclerosis patients, upper motor neuron function varies over time and consequently, spasticity is less predictable compared to post-stroke patients. In patients with multiple sclerosis, spasticity prevails in the lower limbs, while in patients with stroke upper limb spasticity is often predominant. All these differences, which reflect different pathogenetic mechanisms, may help explain the different effects of cannabinoids on spasticity due to stroke compared to the effect on spasticity due to multiple sclerosis.

### Limitations

The present study is monocentric: this can be considered both a limitation and a point of strength. Of course, a multicentre trial would have allowed recruiting a larger number of participants and extrapolating data from a more heterogeneous study population to the overall population of patients with post-stroke spasticity. However, an advantage of our study is that inter-examiner variability is absent since the same examiner consistently performed all assessments including neurophysiological acquisitions.

The limited number of participants is a major limitation of this study and, apart from the mere sample size, a significant beneficial effect of nabiximols could have emerged if more patients with higher baseline levels of pain and spasticity had been recruited.

No functional outcomes have been assessed in this study because we preferred to focus on stretch reflex assessment as the most appropriate sensitive and quantitative measure of spasticity. We believe that stretch reflex can more easily be modulated in the short term by a pharmacological treatment compared to functional assessments.

Indeed, the short duration of the assessment period (1-month treatment including titration) is a limitation of the study. Conceivably, a longer treatment period, possibly associated with a specific rehabilitative treatment could have determined a larger improvement of spasticity and related functional outcomes.

## Conclusions

In conclusion, the main result of this study is the acceptable safety profile of nabiximols in patients with post-stroke spasticity. More studies involving patients with post-stroke spasticity and higher levels of pain are warranted to assess nabiximols effect on both pain and spasticity. Moreover, our findings promote investigations in human subjects concerning a possible beneficial effect of cannabinoids on stroke prevention and trigger neuroprotective mechanisms during the acute phase, as suggested by preclinical studies.

## Data Availability Statement

The raw data supporting the conclusions of this article will be made available by the authors, without undue reservation.

## Ethics Statement

The studies involving human participants were reviewed and approved by Comitato Etico Regione della Liguria. The patients/participants provided their written informed consent to participate in this study.

## Author Contributions

LMa conceived and designed the study, collected data as principal investigator, performed statistical analyses, and drafted the manuscript. LP and LMo performed patients evaluation and collected data. ML supervised data analysis and revised the manuscript. GR performed the cardiological evaluation and revised the manuscript. AC, FF, and CT critically revised the work for important intellectual content. All authors contributed to the article and approved the submitted version.

## Funding

This was an investigator-initiated study, the insurance policy covering the study was provided by Almirall Group, the drug samples (nabiximols and placebo) packaged for the double-blind condition were provided free of charge by GW Pharma. Almirall Group also contributed partially to the total costs of the research, providing the devices for blood pressure and heart rate measurements, covering the costs of consumable materials and partially compensating for research time (40% of total costs - LMa). The funders were not involved in the study design, collection, analysis, interpretation of data, the writing of this article or the decision to submit it for publication.

## Conflict of Interest

Unrelated to this project, Almirall Italia provided research funding to CT. The remaining authors declare that the research was conducted in the absence of any commercial or financial relationships that could be construed as a potential conflict of interest.

## Publisher's Note

All claims expressed in this article are solely those of the authors and do not necessarily represent those of their affiliated organizations, or those of the publisher, the editors and the reviewers. Any product that may be evaluated in this article, or claim that may be made by its manufacturer, is not guaranteed or endorsed by the publisher.
